# Supplementary material for: Meis1 establishes the pre-hemogenic endothelial state prior to Runx1 expression
Source: Nat Commun. 2023 Jul 27;14:4537. doi: 10.1038/s41467-023-40283-0 (PMC10374625; doi:10.1038/s41467-023-40283-0)
Supplement: Supplementary file 1 — Supplementary Information [file 41467_2023_40283_MOESM1_ESM.pdf]

# *Meis1* establishes the prehemogenic endothelial state prior to *Runx1* expression

**Authors:** Patrick Coulombe[1,2], Grace Cole[1,3], Amanda Fentiman[1,4], Jeremy D K Parker[1], Eric Yung[5], Misha Bilenky[1], Lemlem Degefie[1], Patrick Lac[1], Maggie YM Ling[1], Derek Tam[1], R Keith Humphries[5, 6], Aly Karsan[1,2,3,4] *

[1]Michael Smith Genome Sciences Centre, BC Cancer, 675 West 10th Avenue, Vancouver, BC, Canada, V5Z 1L3, [2]Department of Experimental Medicine, University of British Columbia, Vancouver, BC, Canada, V6T 2B5, [3]Department of Pathology and Laboratory Medicine, University of British Columbia, Vancouver, BC, Canada, V6T 2B5, [4]Interdisciplinary Oncology Program, University of British Columbia, Vancouver, BC, Canada, V6T 2B5, [5]Terry Fox Laboratory, BC Cancer, 675 West 10th Avenue, Vancouver, BC, Canada, V5Z 1L3, [6]Department of Medical Genetics, University of British Columbia, Vancouver, BC, Canada, V6T 2B5,

* Author for correspondence:
Aly Karsan
Genome Sciences Centre
BC Cancer Agency
675 West 10th Ave
Vancouver, BC V5Z 1L3
Canada
Email: akarsan@bcgsc.ca
Tel: 604-675-8034

**SUPPLEMENTARY INFORMATION**

**Supplementary Table 1: Cell distribution across CITE-seq cluster by genotype**

| Sample | # of cells | Median reads per cell (RNA) | Median genes per cells (RNA) | Median ADT reads per cell | Mean ADT reads per cell |
|---|---|---|---|---|---|
| WT_1 | 1537 | 26,572 | 5,144 | 220 | 546 |
| WT_2 | 1523 | 28,442 | 5,153 | 243 | 614 |
| WT_3 | 651 | 28,553 | 5,118 | 85 | 186 |
| WT_4 | 2598 | 27,641 | 5,080 | N/A | N/A |
| WT_5 | 2114 | 12,720 | 3,421 | 32 | 177 |
| Meis1-flox/VE-cre_1 | 2232 | 11,772 | 3,373 | 41 | 195 |
| Meis1-flox/VE-cre_2 | 2452 | 9,611 | 2,995 | 32 | 271 |
| Meis1-flox/VE-cre_3 | 2061 | 14,895 | 3,683 | 14 | 65 |

**Supplementary Table 2: Cell distribution across CITE-seq cluster by genotype**

| Cluster | # WT cells | # cKO cells | Total # cells | Ratio WT/total | Ratio cKO/total | Observed difference |
|---|---|---|---|---|---|---|
| vEC_1 | 1599 | 1254 | 2853 | 0.560 | 0.440 | -0.121 |
| vEC_2 | 1562 | 1144 | 2706 | 0.577 | 0.423 | -0.154 |
| vEC_3 | 1428 | 1058 | 2486 | 0.574 | 0.426 | -0.149 |
| vEC_4 | 815 | 557 | 1372 | 0.594 | 0.406 | -0.188 |
| aEC | 888 | 624 | 1512 | 0.587 | 0.413 | -0.175 |
| pre-HE | 171 | 77 | 248 | 0.690 | 0.310 | -0.379 |
| EHT | 326 | 162 | 488 | 0.668 | 0.332 | -0.336 |
| EMP | 390 | 357 | 747 | 0.522 | 0.478 | -0.044 |
| Myeloid | 1052 | 1345 | 2397 | 0.439 | 0.561 | 0.122 |
| Megakaryocytes | 192 | 167 | 359 | 0.535 | 0.465 | -0.070 |
| **Total** | 8423 | 6745 | 15168 | 0.555 | 0.445 | -0.111 |

**Supplementary Table 3: Primer list**

| Primers for Genotyping | | |
|---|---|---|
| **Target** | **Forward Primer (5'-3')** | **Reverse Primer (5'-3')** |
| Cre | GTTTCGAATTTACTGACCG | CGCCGCATAACCAGTGAAAC |
| GFP | CACATGAAGCAGCACGACTT | TGCTCAGGTAGTGGTTGTCG |
| Meis1-flox | CCAAAGTAGCCACCAATATCATGA | AGCGTCACTTGGAAAAGCAATGAT |
| Floxed allele | AGCTTCATTTGAAGTTCCCTATTG | TATTAGGTGGATCCAAGCTTCATT |
| Excised allele | CTGGACTTTCTCCTTTAGTTGGAT | GGAACTTCATCAGTCAGGTACATA |
| **Primers for Gene Expression** | | |
| **Gene** | **Forward Primer (5'-3')** | **Reverse Primer (5'-3')** |
| Meis1 | ATATCATGAGGGCGTGGCTG | CCTGTATCTTGTGCCAACTGC |
| Runx1 | GCCTAGAAGAACCAGGTAGC | TGGTAGGTGGCAACTTGTGG |
| Gfi1 | CCTGGTCAAGAGCAAGAAGG | CTCCATTTTCGACTCGCCTG |
| Spi1 | AACAGATGCACGTCCTCGATA | CATCCGGGGCATGTAGGAA |
| Foxp1 | GGTCTGAGACAAAAAGTAACGGA | CGCACTCTAGTAAGTGGTTGC |
| Pbx1 | CAGCGGGTTCTTCCAGTTCTT | CGAGTCCGTCACTGTATCCTC |
| Smad6 | GCAACCCCTACCACTTCAGC | GTGGCTTGTACTGGTCAGGAG |
| Vegfc | GAGGTCAAGGCTTTTGAAGGC | CTGTCCTGGTATTGAGGGTGG |
| Tulp4 | TTTGCAGCGATTCCAACATCC | GCCAACCATCCCTCTTCATAGT |

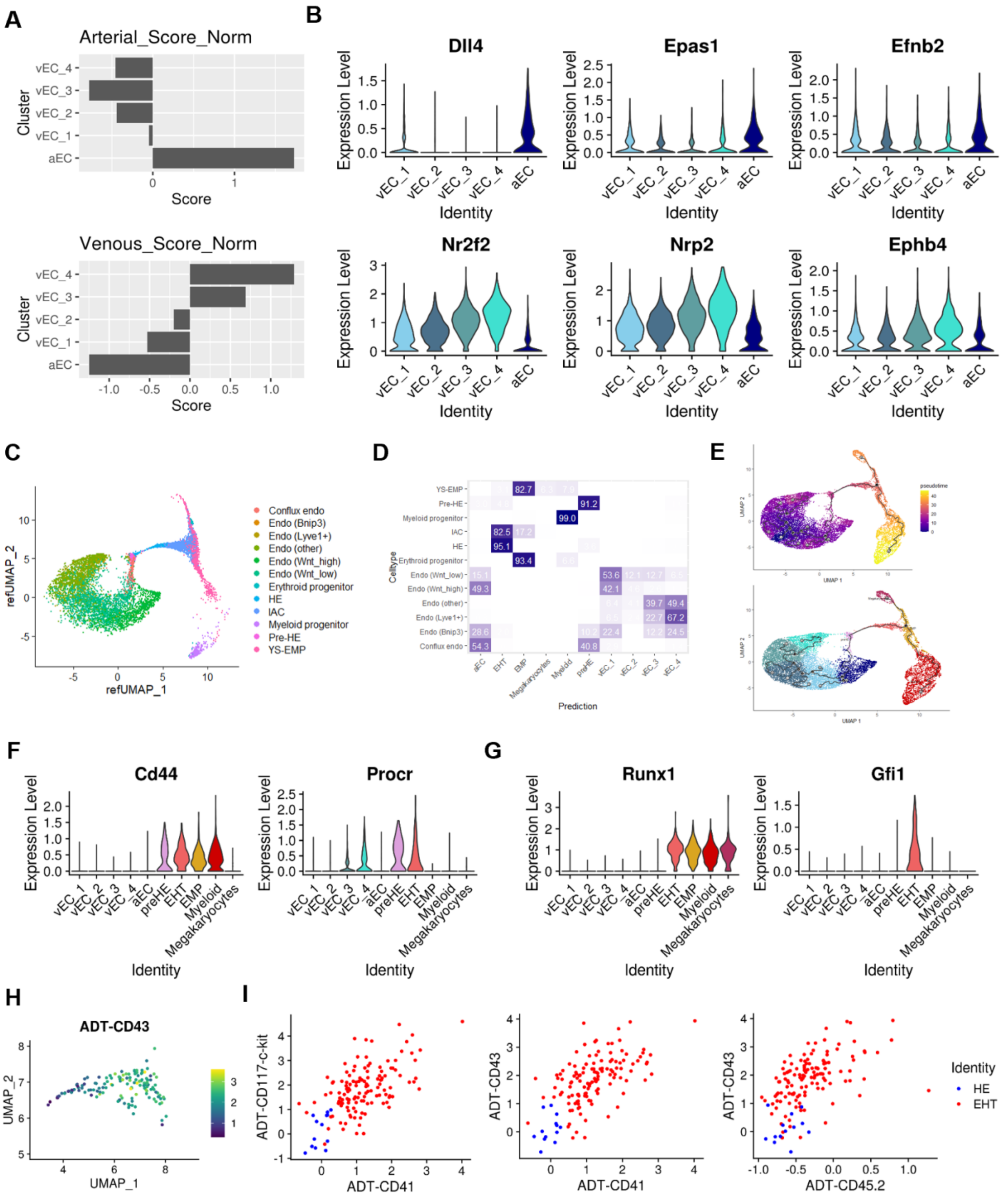


**Supplementary Fig. 1: Cluster identification based on key gene expression**

**(A)** Arterial and venous score for each EC cluster. Scores were based on the mean expression of combined arterial (*Dll4*, *Notch1*, *Notch4*, *Gja5*, *Nrp1*, *Jag1*, *Efnb2*, *Epas1*, *Vegfc*) or venous (*Nr2f2*, *Nrp2*, *Ephb4*) genes, normalized using the Z-score approach. **(B)** Expression of selected

arterial and venous endothelial genes, shown in violin plot for each EC cluster. **(C)** Projection of single-cells from Zhu et al. (2020) dataset onto the UMAP in Fig. 1A. Cells are colored based on their cell type labeling in Zhu et al. **(D)** Correlation matrix showing the annotation of single-cells from Zhu et al. (y-axis) as they are projected onto the clusters from the current paper (x-axis). Frequency is expressed as a percentage of the cluster in Zhu et al. **(E)** Trajectory analysis of clusters during EHT using monocle3 on the CITE-seq dataset; with cells colored by pseudotime (top) and by cell population (bottom). **(F-G)** Violin plot showing the expression of **(F)** pre-HE and **(G)** HE genes from the CITE-seq transcriptomic data. **(H)** Progressive upregulation of CD43 surface expression, based on ADT, shown for the EHT subset. **(I)** Expression of pre-HSPC surface markers, based on ADT, on cells forming the "EHT" subpopulation of the CITE-seq dataset. Cells negative for all the pre-HSPC markers are highlighted in blue and defined as "HE".

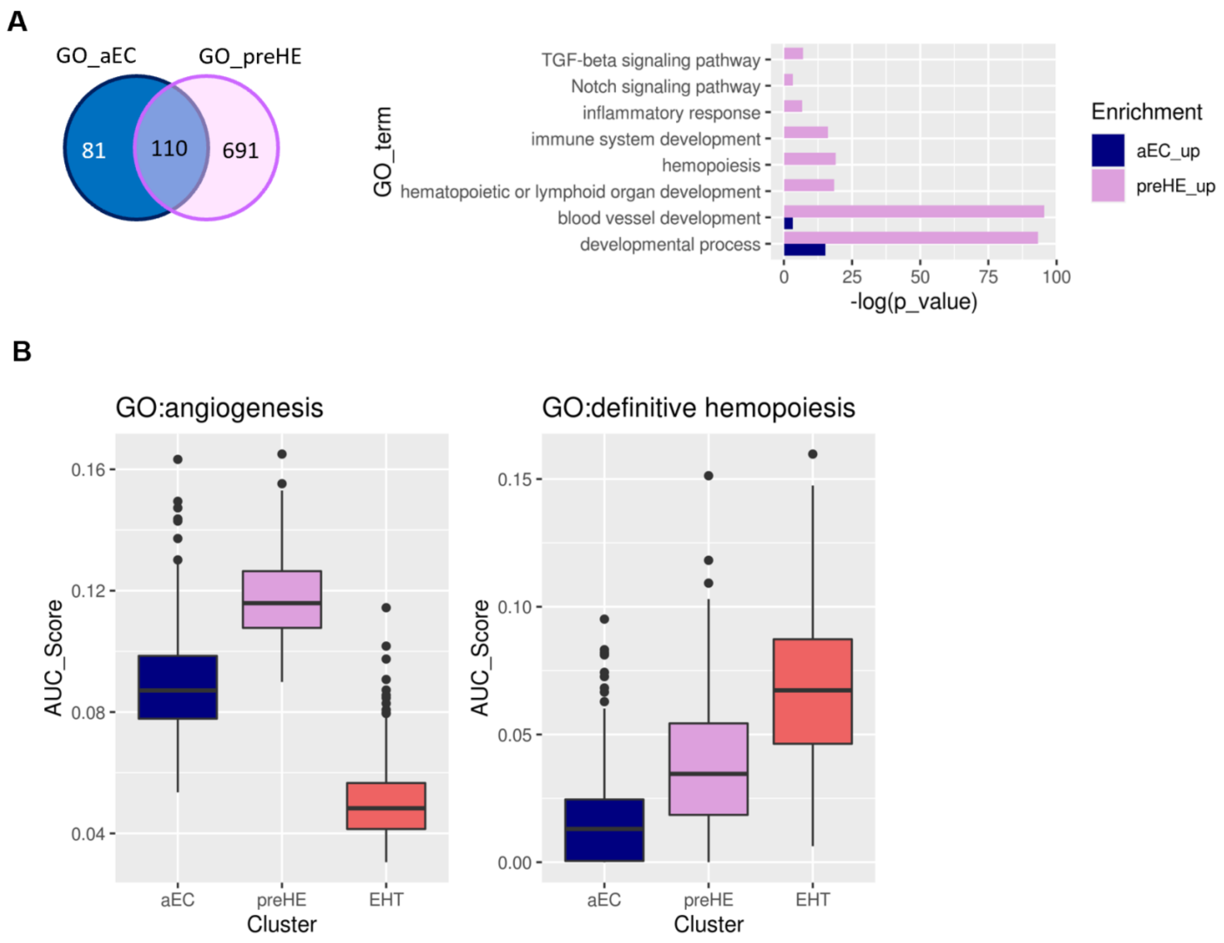


**Supplementary Fig. 2: Characterization of the pre-HE cluster based on gene ontology**

**(A)** Venn diagram depicting the number of unique GO terms enriched in aEC and pre-HE cells and significance of the enrichment (-log(p-value)) for selected GO terms (hypergeometric p-value with g:SCS correction in gprofiler2). **(B)** AUCell scores for the expression of genes associated with selected GO term, in individual cells of the aEC, pre-HE, and EHT clusters. Box-plots display the median (center line), the $25^{th}$ and $75^{th}$ percentiles (box limits), and ±1.5*interquartile range (whiskers). N = 888 aEC, 171 preHE, and 326 EHT cells from 5 independent WT samples.

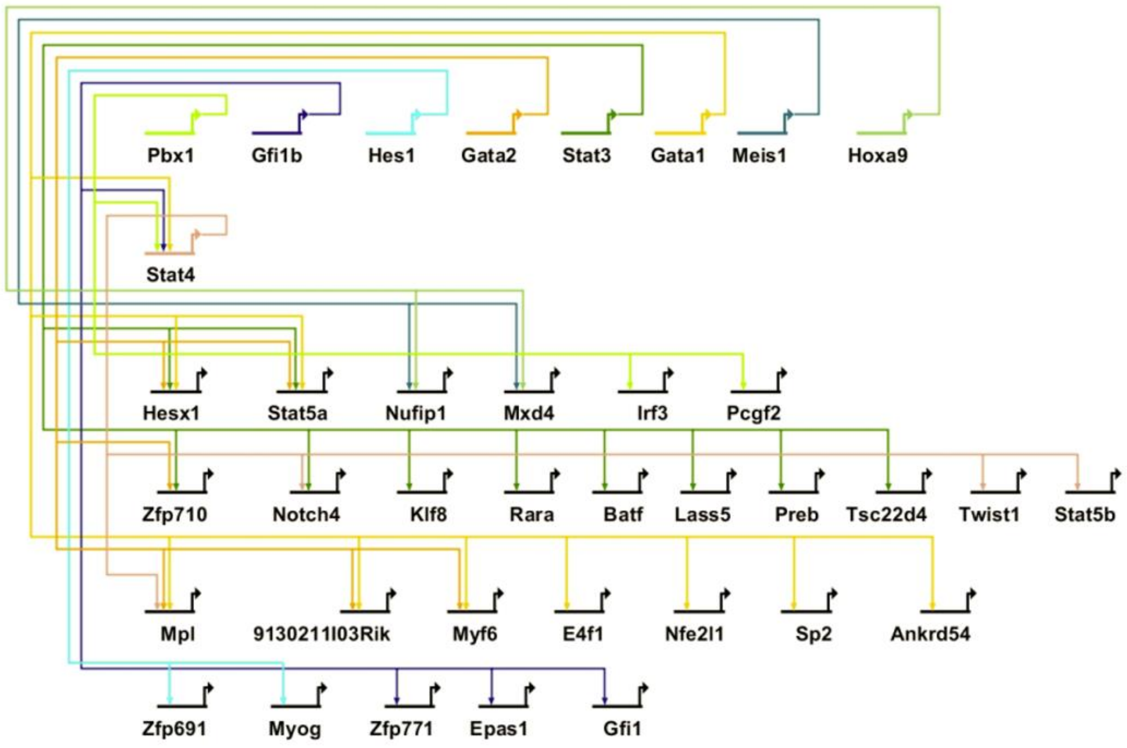


**Supplementary Fig. 3: Predicted TF hierarchy in hemogenic cells at E10.5**

Hierarchy displaying relationships between the 228 up-regulated TFs in our RNA-seq, as predicted by DiRE and visualized in BioTapestry.

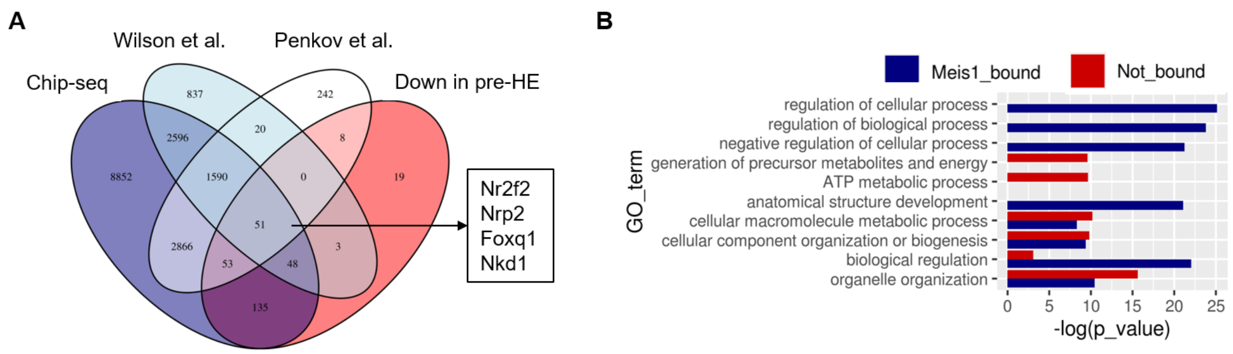


**Supplementary Fig. 4: Meis1 potential repression of aEC genes**

**(A)** Overlapping genes between Meis1 ChIP-seq datasets and genes downregulated in pre-HE vs aEC in the CITE-seq data. **(B)** Top GO terms associated with pre-HE downregulated genes categorized based on the proximity of Meis1 binding sites in ChIP-seq data (hypergeometric p-value with g:SCS correction in gprofiler2).

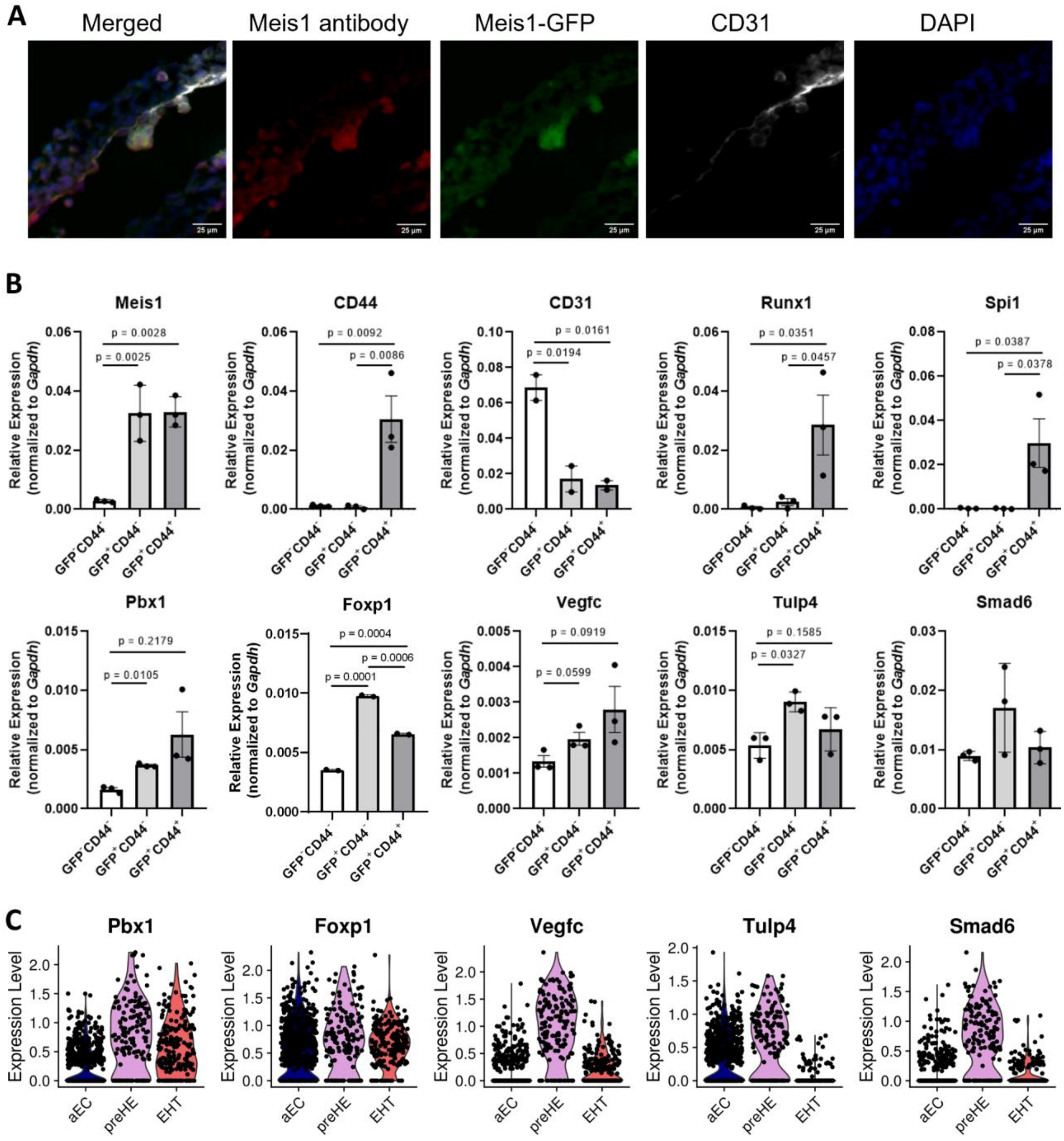


**Supplementary Fig. 5: Meis1$^{+}$ EC show upregulation of early pre-HE genes prior to expression of CD44**

**(A)** Validation of the Meis1-GFP reporter mouse by immunostaining of a E10.5 AGM using Meis1/2 sc10599 antibody (1:200). 40X magnification are shown (scale bar = 25µm). N = 2 independent experiments. **(B)** mRNA expression of selected putative Meis1 target genes in FACS-isolated GFP$^{-}$CD44$^{-}$ EC, GFP$^{+}$CD44$^{-}$ EC and GFP$^{+}$CD44$^{+}$ populations from E9.5 AGM of Meis1-GFP embryos. Genes were considered to be potential Meis1 targets if they were identified in all three ChIP-seq datasets, upregulated during the aEC to pre-HE transition in

CITE-seq dataset, and enriched in Meis1-OE EC vs YFP-CTRL EC in the RNA-seq dataset. ddPCR was performed 3 times independently. For each replicate cells were pooled from two separate litters to harvest enough material for analysis. In total 28 embryos were used for analysis. Quantification was performed by ddPCR and normalized to *Gapdh*. Data are presented as mean ± SEM (p-values based on two-sided t-test). **(C)** Expression of selected genes is enriched in pre-HE population at E10.5 based on CITE-seq data analysis. Source data are provided as a Source Data file.

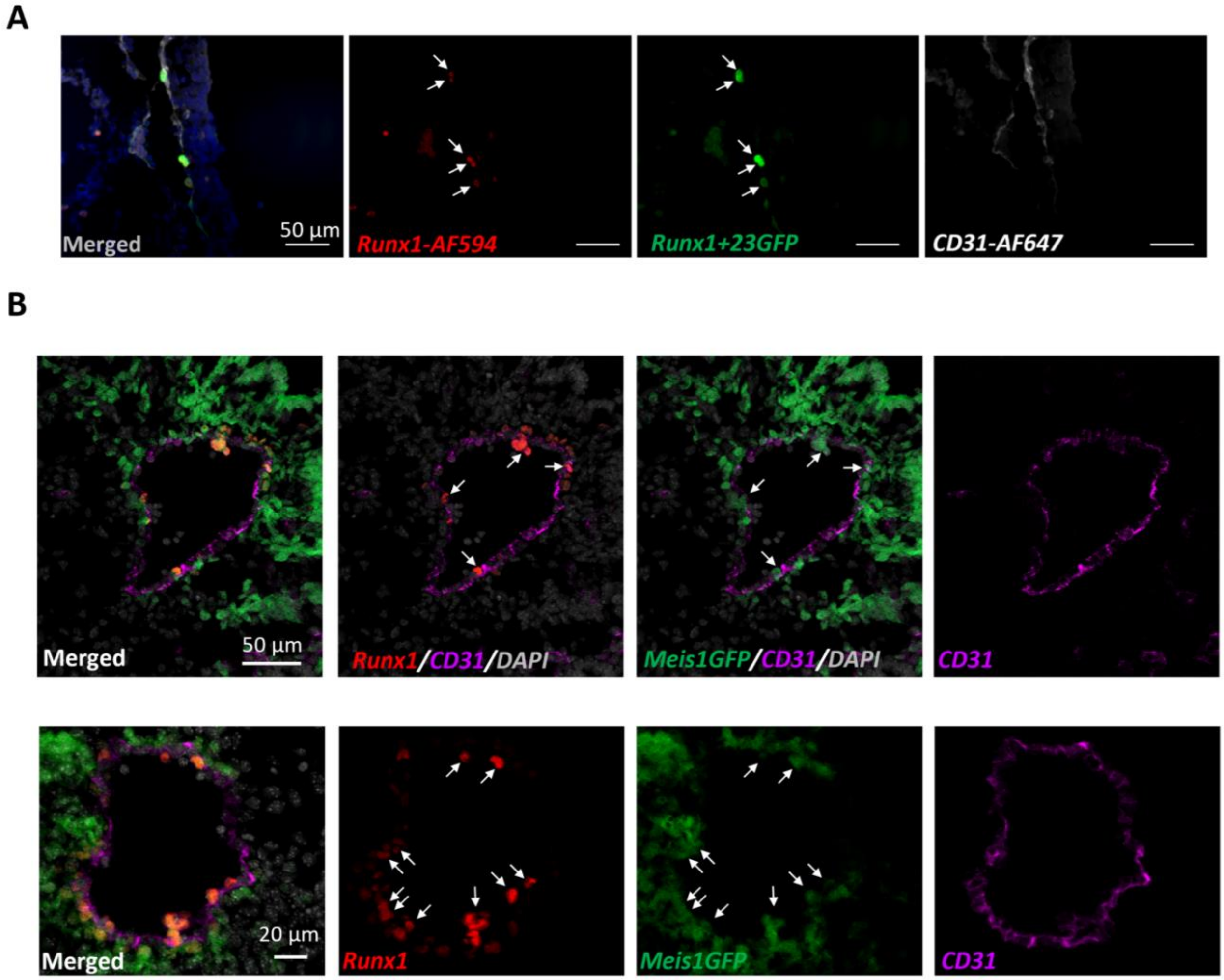


**Supplementary Fig. 6: Immunostaining of Runx1 in E10.5 AGM**

**(A)** Validation of Runx1 antibody in Runx1+23GFP reporter mouse. Runx1 antibody staining (Abcam #ab92336; 1:200 dilution) detects the same cells as GFP staining (green) in Runx1+23GFP embryos. White arrows point at Runx1-expressing cells (scale bar = 50µm; n = 1 embryo). **(B)** Co-immunostaining of Runx1 (red), Meis1 (green), and CD31 (purple) in the dorsal aorta of Meis1-GFP embryos at E10.5 (see also Fig. 4E). White arrows point at endothelial cells co-expressing Meis1 and Runx1. 2 independent experiments were performed. Scale bar = 50µm (upper panels) and 20µm (lower panels).

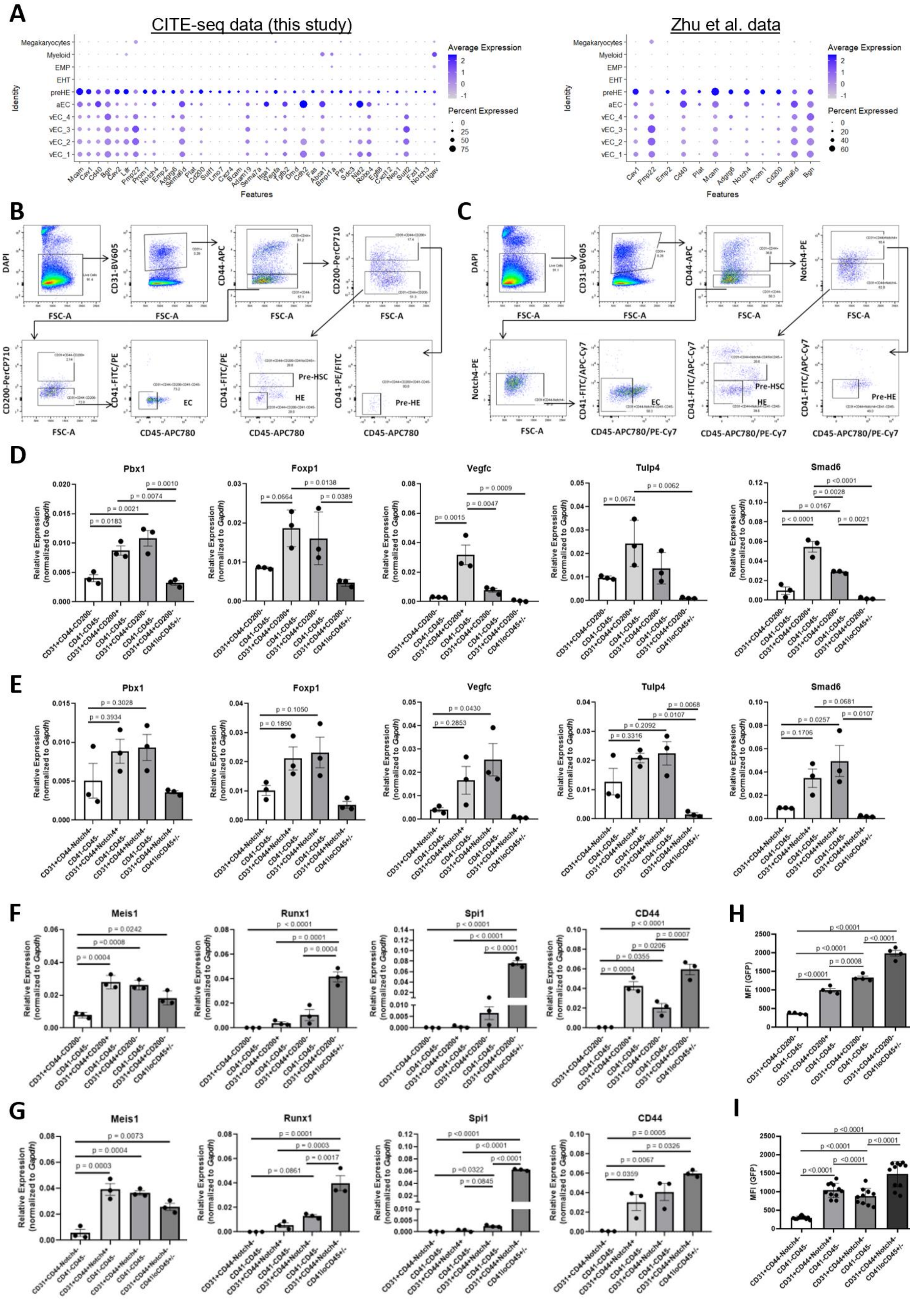
A
CITE-seq data (this study)
Zhu et al. data
Megakaryocytes
Myeloid
EMP
EHT
preHE
aEC
vEC_4
vEC_3
vEC_2
vEC_1
Identity
Features
Average Expression
Percent Expressed
B
C
DAPI
FSC-A
CD31-BV605
CD44-APC
CD200-PerCP710
Notch4-PE
CD41-FITC/PE
CD41-PE/FITC
CD41-FITC/APC-Cy7
CD45-APC780
CD45-APC780/PE-Cy7
EC
HE
Pre-HSC
Pre-HE
D
Pbx1
Foxp1
Vegfc
Tulp4
Smad6
Relative Expression (normalized to Gapdh)
E
F
Meis1
Runx1
Spi1
CD44
G
H
I
MFI (GFP)

**Supplementary Fig. 7: CD200 and Notch4 distinguish pre-HE from HE cells.**

**(A)** We identified cell surface markers expressed highly within the pre-HE population, but having little to no expression on cells undergoing EHT within our CITE-seq dataset and the Zhu et al. dataset (onto which we applied our cell annotation to match the same populations). We screened the list of candidate cell surface markers for low expression on endothelial populations and antibody availability. **(B-C)** Representative flow cytometry plots of the gating strategy to isolate pre-HE based on **(B)** CD200 and **(C)** Notch4. For each marker, we isolated EC ($CD31^{+}CD44^{-}Marker^{-}CD41^{-}CD45^{-}$), pre-HE cells ($CD31^{+}CD44^{+}Marker^{+}CD41^{-}CD45^{-}$), HE cells ($CD31^{+}CD44^{+}Marker^{-}CD41^{-}CD45^{-}$), and pre-HSPC ($CD31^{+}CD44^{+}Marker^{-}CD41^{lo}CD45^{-/+}$), to examine expression of genes of interest. **(D-G)** mRNA was extracted from FACS-isolated populations from E10.5 AGM to assess, by ddPCR, the expression of selected putative Meis1 target genes expressed in pre-HE **(D-E)** and HE hematopoietic genes **(F-G)**. ddPCR was performed 3 times independently. For each replicate, cells were pooled from two separate litters to harvest enough material for analysis. In total 30 and 36 embryos were used for analysis of CD200 **(D, F)** and Notch4 **(E, G)** purified populations, respectively. Quantification was performed by ddPCR and normalized to *Gapdh*. **(H-I)** Mean fluorescence intensity of Meis1GFP in the gated populations of EC, pre-HE, HE, and pre-HSPC by flow cytometry based on **(H)** CD200 and **(I)** Notch4. **(H)** N = a total of 4 embryos from 2 independent litter and **(I)** n = a total of 11 embryos from 3 independent litter. For all bar plots, data are presented as mean ± SEM (p-values based on one-way ANOVA). Source data are provided as a Source Data file.

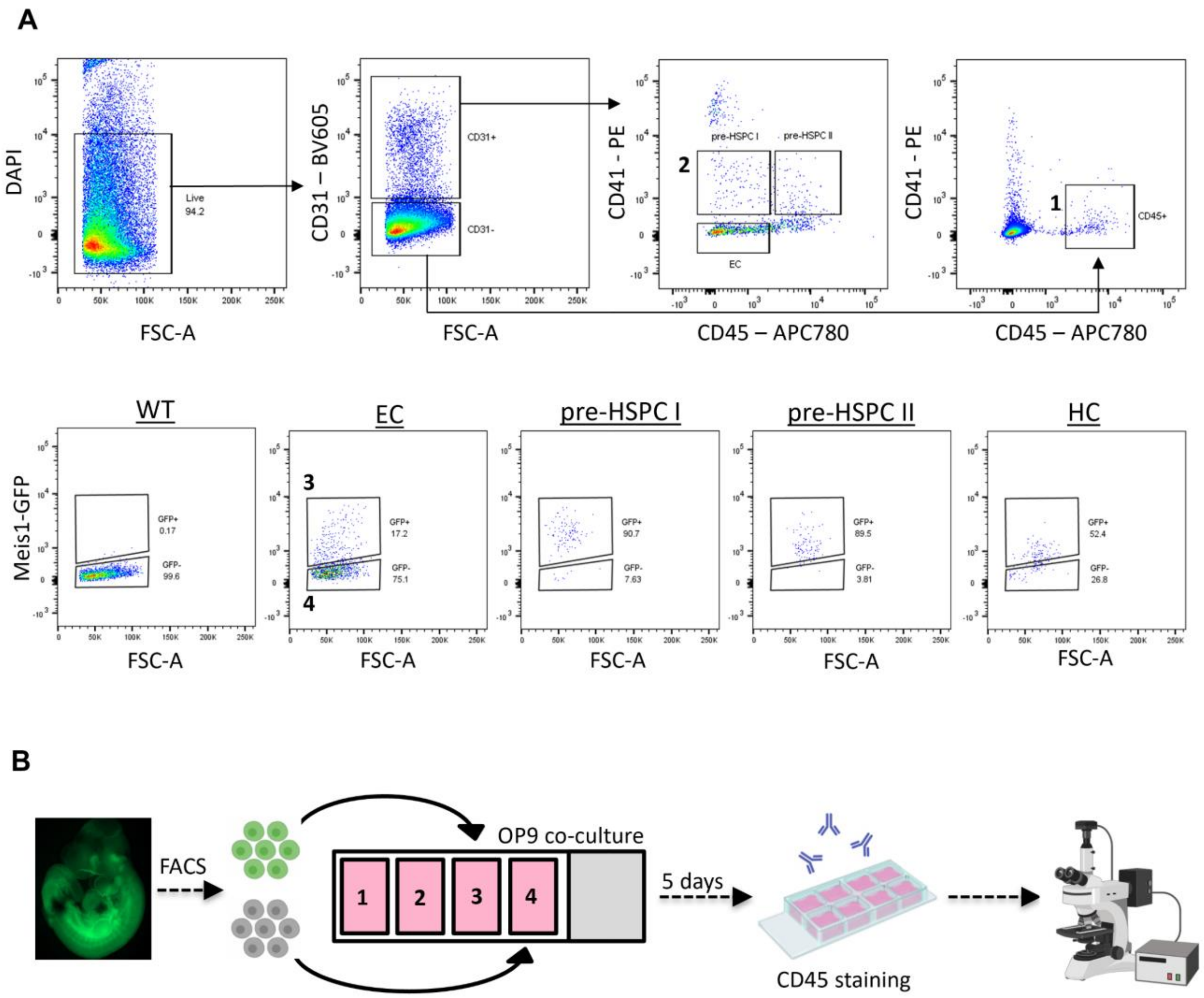


**Supplementary Fig. 8: FACS strategy for Meis1-GFP embryos and *ex vivo* experiments**

**(A)** Representative flow cytometry plot showing the gating strategy for EC, pre-HSPC I, pre-HSPC II, and HC as well as GFP expression in each subpopulation. **(B)** Schematic of the *ex vivo* co-culture assay with OP9 to asses hemogenic potential in GFP+ and GFP- EC. Four cell fractions were FACS-isolated (gates labeled 1-4 in panel A) to include controls for the experiment. Schematic was partially created with BioRender.com

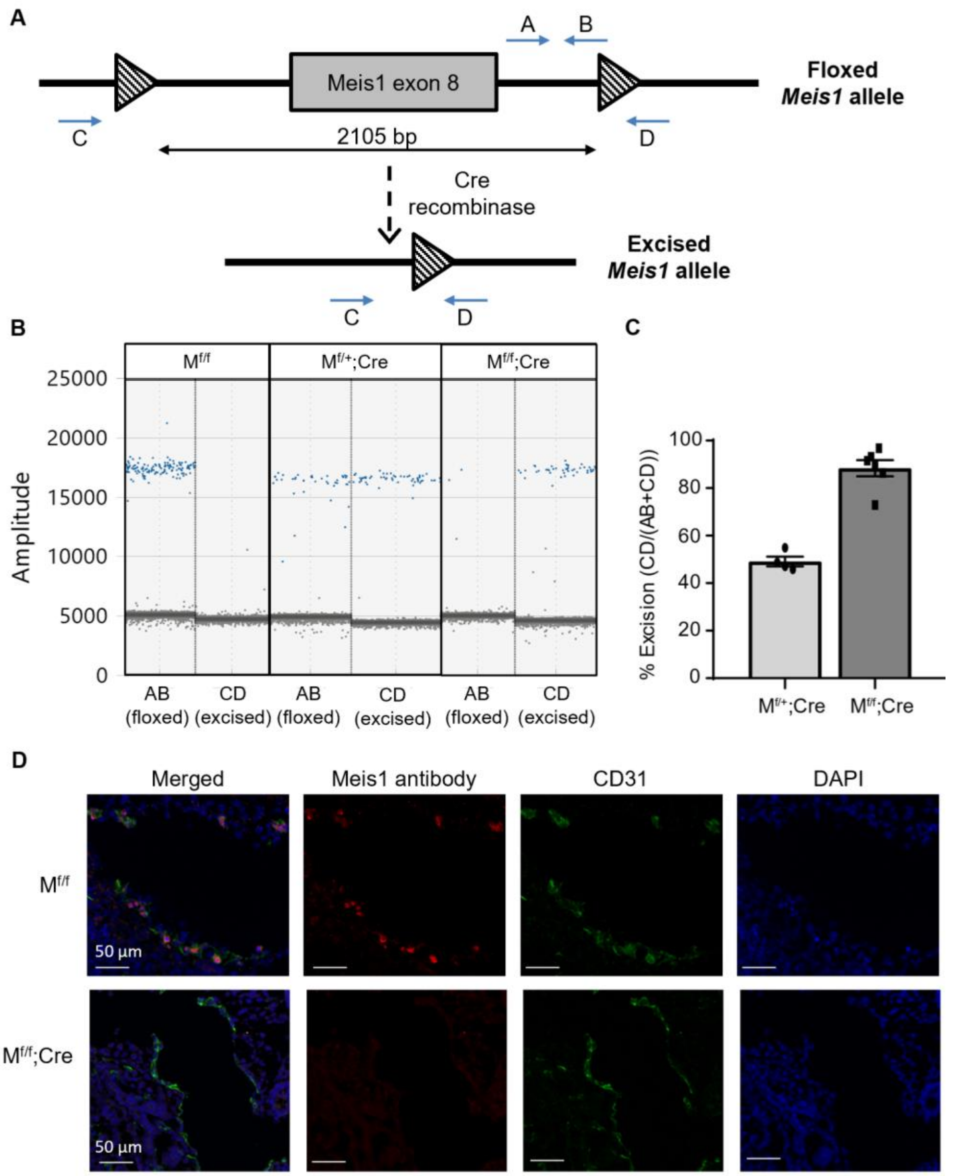


**Supplementary Fig. 9: Quantification of *Meis1* locus excision in VEC-Cre cells**

Genomic DNA was extracted from CD31$^{+}$ EC FACS-isolated from E10.5 AGM to assess, by ddPCR, the efficiency of Cre recombination. **(A)** Schematic of the *Meis1* floxed allele at exon 8, before and after Cre recombination. Arrows depict the location of primers used for PCR amplification. **(B)** Representative ddPCR plot to quantify the presence of the floxed allele before (primer AB) and after recombination (primer CD). **(C)** Quantification of the excised amplicon detected by ddPCR, as a percentage of the total allele cDNA (floxed + excised). Data are presented as mean ± SEM (n = 4 $M^{f/+}$;Cre and 6 $M^{f/f}$;Cre). **(D)** Immunostaining of $M^{f/f}$ (top) and $M^{f/f}$;Cre (bottom) AGM to validate deletion of the Meis1 protein product in the conditional knockout embryos at E10.5. Scale bar = 50µm. Staining was performed in 2 independent experiments (n = 2 embryos for each condition)

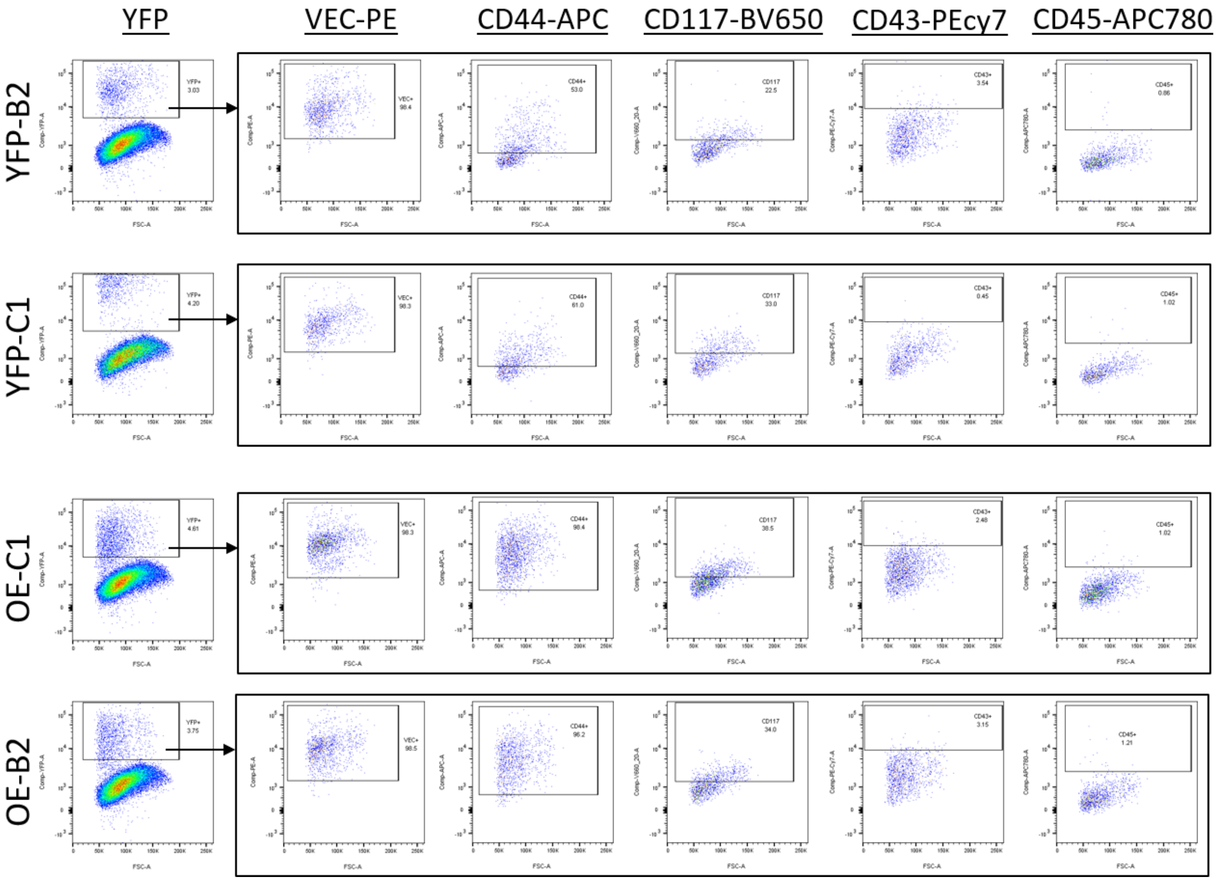


**Supplementary Fig. 10: Flow cytometry analysis of Meis1-OE EC after co-culture with OP9.**

Representative flow cytometry plots of the expression of selected surface markers on Meis1-OE and YFP-CTRL EC after co-culture with OP9 cells. Plots show expression on gated YFP+ cells (left panels) to remove OP9 cells.
