# Supplementary material for: Meis1 establishes the pre-hemogenic endothelial state prior to Runx1 expression
Source: Nat Commun. 2023 Jul 27;14:4537. doi: 10.1038/s41467-023-40283-0 (PMC10374625; doi:10.1038/s41467-023-40283-0)
Supplement: Supplementary file 3 — Reporting Summary [file 41467_2023_40283_MOESM3_ESM.pdf]

nature portfolio

Corresponding author(s): Aly Karsan

Last updated by author(s): Jun 15, 2023

Reporting Summary

Nature Portfolio wishes to improve the reproducibility of the work that we publish. This form provides structure for consistency and transparency in reporting. For further information on Nature Portfolio policies, see our Editorial Policies and the Editorial Policy Checklist.

Statistics

For all statistical analyses, confirm that the following items are present in the figure legend, table legend, main text, or Methods section.

n/a	Confirmed	
☐	☒	The exact sample size (*n*) for each experimental group/condition, given as a discrete number and unit of measurement
☐	☒	A statement on whether measurements were taken from distinct samples or whether the same sample was measured repeatedly
☐	☒	The statistical test(s) used AND whether they are one- or two-sided<br>*Only common tests should be described solely by name; describe more complex techniques in the Methods section.*
☒	☐	A description of all covariates tested
☐	☒	A description of any assumptions or corrections, such as tests of normality and adjustment for multiple comparisons
☐	☒	A full description of the statistical parameters including central tendency (e.g. means) or other basic estimates (e.g. regression coefficient) AND variation (e.g. standard deviation) or associated estimates of uncertainty (e.g. confidence intervals)
☐	☒	For null hypothesis testing, the test statistic (e.g. *F*, *t*, *r*) with confidence intervals, effect sizes, degrees of freedom and *P* value noted<br>*Give P values as exact values whenever suitable.*
☒	☐	For Bayesian analysis, information on the choice of priors and Markov chain Monte Carlo settings
☒	☐	For hierarchical and complex designs, identification of the appropriate level for tests and full reporting of outcomes
☒	☐	Estimates of effect sizes (e.g. Cohen's *d*, Pearson's *r*), indicating how they were calculated

Our web collection on statistics for biologists contains articles on many of the points above.

Software and code

Policy information about availability of computer code

Data collection	CITE-seq data was sequenced on Illumina NextSeq500 or NextSeq2000. Illumina RTA software used for basecalling and cellranger count was ran using cellranger version 3.0.2. For bulk RNA-seq, samples were sequenced on Illumina HiSeq 2000 with corresponding Illumina RTA software. Co-expression data was obtained through the online tool EnrichR (https://maayanlab.cloud/Enrichr/). Flow cytometry data were acquired using BD FACSDiva software (v.8.0.1). ddPCR was performed on a BioRad QX200 instrument using Quantasoft. Microscopy image were capture using ZEN 3.2 software.
Data analysis	FACS data were analyzed using FlowJo (v10). Gene expression mesured by ddPCR was analyzed using BioRad Quantasoft (v1.7.4). CITE-seq data was analyzed in R (version 3.6.3) using the following available packages: Seurat (v3), AUCell (v1.14.0), and scProportionTest (v0.0.0.9000). Gene ontology analysis was performed in R using gprofiler2 (v0.2.1). The online Genomic Regions Enrichment of Annotations Tool (GREAT v4; http://great.stanford.edu/public/html/) was used to map ChIP-seq peaks to genes. Differential gene expression analysis of Meis1-OE RNA-seq was performed in R using DESeq2 (v1.32.0). GraphPad Prism 7.00 was also used for some statistical analysis. DiRE (https://dire.dcode.org/) was used to analyze gene co-regulation. BWA v0.7 (https://bio-bwa.sourceforge.net/index.shtml) was used to align the ChIP-seq data.

For manuscripts utilizing custom algorithms or software that are central to the research but not yet described in published literature, software must be made available to editors and reviewers. We strongly encourage code deposition in a community repository (e.g. GitHub). See the Nature Portfolio guidelines for submitting code & software for further information.

Data

Policy information about availability of data

All manuscripts must include a data availability statement. This statement should provide the following information, where applicable:

- Accession codes, unique identifiers, or web links for publicly available datasets
- A description of any restrictions on data availability
- For clinical datasets or third party data, please ensure that the statement adheres to our policy

Raw data files for sequencing studies were The CITE-seq data, the RNA-seq data for HE cells, and the RNA-seq data for Meis1-OE cells have been deposited in the NCBI Gene Expression Omnibus and are available from the following accession numbersunder accession code: GSE197244 [https://www.ncbi.nlm.nih.gov/geo/query/acc.cgi?acc=GSE197244], GSE196047 [https://www.ncbi.nlm.nih.gov/geo/query/acc.cgi?acc=GSE196047], GSE197244, and GSE197400 [https://www.ncbi.nlm.nih.gov/geo/query/acc.cgi?acc=GSE197400], respectively. The ChIP-seq data have been deposited in the European Nucleotide Archive under accession code PRJEB52790 [https://www.ebi.ac.uk/ena/browser/view/PRJEB52790]. Co-expression data for the Meis1 analysis can be downloaded from the EnrichR webserver (https://maayanlab.cloud/Enrichr/). The published ChIP-seq datasets used in this study were downloaded from the original publications [17, 18]. Source data are provided with this paper.

Research involving human participants, their data, or biological material

Policy information about studies with human participants or human data. See also policy information about sex, gender (identity/presentation), and sexual orientation and race, ethnicity and racism.

Reporting on sex and gender	N/A
Reporting on race, ethnicity, or other socially relevant groupings	N/A
Population characteristics	N/A
Recruitment	N/A
Ethics oversight	N/A

Note that full information on the approval of the study protocol must also be provided in the manuscript.

Field-specific reporting

Please select the one below that is the best fit for your research. If you are not sure, read the appropriate sections before making your selection.

☒ Life sciences ☐ Behavioural & social sciences ☐ Ecological, evolutionary & environmental sciences

For a reference copy of the document with all sections, see nature.com/documents/nr-reporting-summary-flat.pdf

Life sciences study design

All studies must disclose on these points even when the disclosure is negative.

Sample size	There was no sample size determination prior to performing the experiments. Experiments were performed using a minimum of 3 independent biological samples to perform statistical analysis. For embryo experiments, at least 3 different litters were collected and analyze to observed data reproducibility.
Data exclusions	For the CITE-seq data, cells with < 2000 genes detected and/or >10% mitochondrial content were excluded from the analysis as a pre-filtering quality control step to include only live cells with good sequencing coverage.<br>One data point was excluded from Figure 3i) based on Grubbs' outlier test (alpha = 0.05). The magnitude of the cell count observed for that sample was unusually high, likely due to an issue with the counting beads (for example: too many beads added during processing)
Replication	A total of 5 WT and 3 Meis1-flox/VE-Cre samples (each representing a pool of embryos) were submitted for CITE-seq and showed great reproducibility based on single-cell clustering analysis. For RNA-seq, endothelial cells were transduced in three independent replicates (for each condition) and each replicate was successfully sequenced (data for all 3 replicates can be found in the manuscript). For all experiments comparing embryos of different genotypes, a minimum of 3 litters were analyzed to ensure reproducibility of the data.
Randomization	Randomization was not performed. All samples were treated the same way for this study.
Blinding	Researchers were blinded to the genotype of embryos during sample processing and data collection. Researchers were not blinded during data nalysis.

Reporting for specific materials, systems and methods

We require information from authors about some types of materials, experimental systems and methods used in many studies. Here, indicate whether each material, system or method listed is relevant to your study. If you are not sure if a list item applies to your research, read the appropriate section before selecting a response.

Materials & experimental systems

n/a	Involved in the study	
☐	☒	Antibodies
☐	☒	Eukaryotic cell lines
☒	☐	Palaeontology and archaeology
☐	☒	Animals and other organisms
☒	☐	Clinical data
☒	☐	Dual use research of concern
☒	☐	Plants

Methods

n/a	Involved in the study	
☐	☒	ChIP-seq
☐	☒	Flow cytometry
☒	☐	MRI-based neuroimaging

Antibodies

Antibodies used

The following antibodies were used for immunostaining: CD31 antibody from BD Biosciences (Cat#550274; RRID:AB_393571), CD45 antibody, Clone 30-F11 (RUO) from BD Biosciences (Cat#550539; RRID:AB_2174426), GFP Polyclonal Antibody, Alexa Fluor 488 from ThermoFisher Scientific (Cat#A-21311; RRID:AB_221477), Runx1 antibody [EPR3099] from Abcam (Cat# ab92336; RRID:AB_2049267), and anti-Meis1/2 from Santa Cruz (cat#sc-10599)
The following FACS antibodies were purchased from BioLegend: APC anti-mouse/human CD44 antibody (Cat#103012; RRID:AB_312963), Brilliant Violet 605™ anti-mouse CD31 [390] (Cat#102427; RRID:AB_2563982), FITC anti-mouse CD41 [MWReg30] (Cat#133904; RRID:AB_2129746), PE anti-mouse CD144 (VE-cadherin) antibody (Cat#138105; RRID:AB_2077941), APC/Cy7 anti-Mouse CD41 [MWReg30] (Cat#133928), and PE/Cy7 anti-mouse CD43 [S11] (Cat#143209)
The following FACS antibodies were purchased from ThermoFisher Scientific: CD41a Monoclonal Antibody (eBioMWReg30(MWReg30)) PE (Cat#12-0411-82; RRID:AB_763485), CD45.2 Monoclonal Antibody (104) APC-eFluor 780 eBioscience™ (Cat#47-0454-82; RRID:AB_1272175), anti-mouse Notch4 [HMN4-14] PE (Cat#12-5764-80), and anti-mouse CD200 [OX90] PerCPe710 (Cat#46-5200-80)
The following CITE-seq antibodies were purchased from BioLegend: TotalSeq™-A0012 anti-mouse CD117 (c-kit) Antibody (Cat#105843; RRID:AB_2749960), TotalSeq™-A0073 anti-mouse/human CD44 Antibody (Cat#103045; RRID:AB_2734154), TotalSeq™-A0110 anti-mouse CD43 Antibody (Cat#143211; RRID:AB_2750541), TotalSeq™-A0157 anti-mouse CD45.2 Antibody (Cat#109853; RRID:AB_2783051), TotalSeq™-A0191 anti-mouse/rat/human CD27 Antibody (Cat#124235; RRID:AB_2750344), TotalSeq™-A0443 anti-mouse CD41 Antibody (Cat#133937; RRID:AB_2800635).
The following antibody were purchased from abcam: anti-HA anti-HA (cat# ab9110).

Validation

Validation data for each of the primary antibodies employed in this study are available on the manufacturer's website, along with citation references and application specific technical details. In addition, the GFP antibody was validated in our lab against tissue samples from wildtype embryos (negative control) and appropriate primary antibodies matching our mouse model (Meis1 or Runx1 antibodies). Flow cytometry antibodies were tested by both BioLegend and ThermoFisher Scientific in comparison to the appropriate isotype control as detailed on their websites. In all our flow cytometry experiments, fluorescence minus one (FMOs) were used to control for background staining. CITE-seq antibodies were validated by BioLegend; each lot of antibody is quality control tested by immunofluorescent staining with flow cytometric analysis and the oligomer sequence is confirmed by sequencing, as described on the manufacturer's website.

Eukaryotic cell lines

Policy information about cell lines and Sex and Gender in Research

Cell line source(s)	OP9 cells from ATCC (Cat# CRL-2749; RRID:CVCL_4398)
Authentication	No authentication was performed
Mycoplasma contamination	OP9 cell line tested negative for mycoplasma
Commonly misidentified lines (See ICLAC register)	None of the cell lines used are listed as commonly misidentified cell lines in the ICLAC register

Animals and other research organisms

Policy information about studies involving animals; ARRIVE guidelines recommended for reporting animal research, and Sex and Gender in Research

Laboratory animals

The following mouse strain were used in this study: Runx1+23mCNE-GFP, Meis1GFP (also named C57BL/6N-Meis1em1Bcca), Meis1-

Laboratory animals	flox, and VEC-Cre (B6;129-Tg(Cdh5-cre)1Spe/J). Males and females of age 6-20 weeks were used in timed mating. Embryos were harvested at 9 or 10 days post-conception (E9.5, E10.5) for experiments.
Wild animals	The study did not involve wild animals.
Reporting on sex	This information has not been collected.
Field-collected samples	The study did not involve samples collected from the field.
Ethics oversight	All animal protocols were approved by the Animal Care Committee of the University of British Columbia (Vancouver, Canada)

Note that full information on the approval of the study protocol must also be provided in the manuscript.

Plants

Seed stocks	*Report on the source of all seed stocks or other plant material used. If applicable, state the seed stock centre and catalogue number. If plant specimens were collected from the field, describe the collection location, date and sampling procedures.*
Novel plant genotypes	*Describe the methods by which all novel plant genotypes were produced. This includes those generated by transgenic approaches, gene editing, chemical/radiation-based mutagenesis and hybridization. For transgenic lines, describe the transformation method, the number of independent lines analyzed and the generation upon which experiments were performed. For gene-edited lines, describe the editor used, the endogenous sequence targeted for editing, the targeting guide RNA sequence (if applicable) and how the editor was applied.*
Authentication	*Describe any authentication procedures for each seed stock used or novel genotype generated. Describe any experiments used to assess the effect of a mutation and, where applicable, how potential secondary effects (e.g. second site T-DNA insertions, mosiacism, off-target gene editing) were examined.*

ChIP-seq

Data deposition

☒ Confirm that both raw and final processed data have been deposited in a public database such as GEO.

☒ Confirm that you have deposited or provided access to graph files (e.g. BED files) for the called peaks.

Data access links<br>*May remain private before publication.*	ttps://www.ebi.ac.uk/ena/browser/view/PRJEB52790
Files in database submission	Meis1+NA10HD_A03268_mm10_xset150_dupsN_ht7.bed.gz
Genome browser session<br>(e.g. UCSC)	https://genome.ucsc.edu/s/pcoulombe/Meis1_A03268_peaks

Methodology

Replicates	Each ChIP-Seq experiment consisted of individual replicates
Sequencing depth	Total number of 36167788 single-end 50bp reads were sequenced on Illumina GAII in Anti-HA (Meis1) ChiP-Seq experiment.
Antibodies	Anti-HA antibody (Abcam cat# ab9110)
Peak calling parameters	FindPeaks 3 tool was used for the analysis. Every aligned single-end read was directionally extended by 150bp (mean fragment length in the ChIP-Seq experiment) and the genome wide fragment coverage profile was calculated. Enriched regions were thresholded using a false discovery rate of ~0.01 (corresponding to the minimal x-coverage height of 7). For the FDR analysis 5 randomization runs were used. Further, peaks boundaries were refined by trimming at coverage equal 30% of their maximal height, in order to remove low height flanks, and separate composite regions. Final list of enrichments for mm10 reference contained 55004 Meis1 bound ChIP-seq regions with average size ~700bp and average peak height ~25.
Data quality	Enriched regions were thresholded using a false discovery rate of ~0.01 (corresponding to the minimal x-coverage height of 7), and refined further by trimming at 30% of their maximal height, in order to remove low height flanks, and separate composite regions.
Software	To identify Meis1 binding regions, Illumina GAII 50-bp-long sequence reads were aligned to the genome (mm9) using Burrows-Wheeler Alignment (BWA):<br>H. Li, R. Durbin<br>Fast and accurate short read alignment with Burrows-Wheeler transform.<br>Bioinformatics 2009 Jul 15;25(14):1754-60. doi: 10.1093/bioinformatics/btp324<br><br>Peak calling (FindPeaks 3):<br>A.P. Fejes, G. Robertson, M. Bilenky, R. Varhol, M. Bainbridge, S.J.M. Jones<br>FindPeaks 3.1: a tool for identifying areas of enrichment from massively parallel short-read sequencing technology<br>Bioinformatics, 2008 Aug 1;24(15):1729–30. doi: 10.1093/bioinformatics/btn305

Flow Cytometry

Plots

Confirm that:

☒ The axis labels state the marker and fluorochrome used (e.g. CD4-FITC).

☒ The axis scales are clearly visible. Include numbers along axes only for bottom left plot of group (a 'group' is an analysis of identical markers).

☒ All plots are contour plots with outliers or pseudocolor plots.

☒ A numerical value for number of cells or percentage (with statistics) is provided.

Methodology

Sample preparation	AGM were dissected from mouse embryos and dissociated in PBS with 2% fetal bovine serum, 1mg/ml collagenase II, and 1mg/ml DNaseI. Cells were resuspended in PBS-2%FBS with DNaseI, blocked 5 minutes with CD16/32 and stained with antibodies for 30 minutes, protected from light.
Instrument	Cells were sorted on a BD FACSAria III or BD FACSAria Fusion instrument
Software	Flow cytometry data was collected using BD FACSDiva Software and analyzed using FlowJo v10.
Cell population abundance	Cell purity post-sort was not assessed given the very small number of cells collected per sample
Gating strategy	For all FACS experiments, the cell population was defined based on FSC-A and SSC-A parameters to remove debris. Doublets were removed based on FSC-H and FSC-W parameters and viable single cells were determined by DAPI staining. Gating for GFP was based on wildtype littermates (used as negative population) and gating for all other fluorochromes was defined based on FMO controls.

☒ Tick this box to confirm that a figure exemplifying the gating strategy is provided in the Supplementary Information.
